# Evaluation of sweet potato for fuel bioethanol production: hydrolysis and fermentation

**DOI:** 10.1186/2193-1801-2-493

**Published:** 2013-09-30

**Authors:** Claudia Lareo, Mario Daniel Ferrari, Mairan Guigou, Lucía Fajardo, Valeria Larnaudie, María Belén Ramírez, Jorge Martínez-Garreiro

**Affiliations:** Depto. Bioingeniería, Facultad de Ingeniería, Universidad de la República, J. Herrera y Reissig 565, CP 11300 Montevideo, Uruguay; Depto. Operaciones Unitarias en Ingeniería Química e Ingeniería de Alimentos, Facultad de Ingeniería, Universidad de la República, J. Herrera y Reissig 565, CP 11300 Montevideo, Uruguay

**Keywords:** Sweet potato, Bioethanol, *Saccharomyces cerevisiae*, Alcoholic fermentation, Simultaneous saccharification and fermentation (SSF)

## Abstract

The enzymatic starch hydrolysis and bioethanol production from a variety of sweet potato developed for bioenergy purposes (K 9807.1) on the basis of its high starch yields, was studied. Drying at 55°C and 95°C of sweet potato neither affected the sugar content nor the starch enzymatic hydrolysis efficiency. Simultaneous saccharification and ethanol fermentations for dry matter ratio of sweet potato to water from 1:8 to 1:2 (w/v) were studied. Fresh sweet potato and dried at 55°C (flour) were assayed. At ratios of 1:8, similar results for fresh sweet potato and flour in terms of ethanol concentration (38–45 g/L), fermentation time (16 h) and sugar conversion (~ 100%) were found. At higher dry matter content, faster full conversion were observed using flour. A higher ratio than that for fresh sweet potato (1:2.2) did not improve the final ethanol concentration (100 g/L) and yields. High ethanol yields were found for VHG (very high gravity) conditions. The sweet potato used is an attractive raw matter for fuel ethanol, since up to 4800 L ethanol per hectare can be obtained.

## Introduction

There is a considerable interest in developing biorenewable alternatives to substitute fossil fuels such as bioethanol as transportation fuel. Bioethanol contributes to diminish petroleum dependency, generates new development opportunities in the agricultural and agro-industrial sectors, more farm work and environmental benefits. Main feedstocks for bioethanol production are sugarcane (Brazil) and corn grain (USA). Because of the increasing demand for ethanol, alternative and non-conventional raw materials are under research (Mussatto et al. 2010).

Sweet potato *(Ipomea batatas*) has been considered a promising substrate for alcohol fermentation since it has a higher starch yield per unit land cultivated than grains (Duvernay et al. 2013; Lee et al. 2012; Srichuwong et al. 2009; Ziska et al. 2009). Industrial sweet potatoes are not intended for use as a food crop. They are bred to increase its starch content, significantly reducing its attractiveness as a food crop when compared to other conventional food cultivars (visual aspect, color, taste). Therefore, they offer potentially greater fermentable sugar yields from a sweet potato crop for industrial conversion processes and the opportunity to increase planted acreage (even on marginal lands) beyond what is in place for food. It has been reported that some industrial sweet potatoes breeding lines developed could produce ethanol yields of 4500–6500 L/ha compared to 2800–3800 L/ha for corn (Duvernay et al. 2013; Ziska et al. 2009). Sweet potato has several agronomic characteristics that determine its wide adaptation to marginal lands such as drought resistant, high multiplication rate and low degeneration of the propagation material, short grow cycle, low illness incidence and plagues, cover rapidly the soil and therefore protect it from the erosive rains and controlling the weed problem (Cao et al. 2011; Duvernay et al. 2013; Vilaró et al. 2009). Previous transformation of the raw material into chips or flour (powder) can be done in order to facilitate its transport and/or plant conservation.

An effective ethanol production process is one where the amount of water added is minimal, since more energy will be required to remove it at the end of the process if the final ethanol concentration is low (Cao et al. 2011; Shen et al. 2011). High ethanol concentration can be reached if the fermentation broth contains high fermentable sugar concentration. In the case of ethanol production from root and tuber crops, it implies the use of a very high gravity (VHG) medium with high solid content and high viscosity. The high viscous nature causes several handling difficulties during processes, and may lead to incomplete hydrolysis of starch to fermentable sugars (Shanavas et al. 2011; Wang et al. 2008; Watanabe et al. 2010; Zhang et al. 2011). Some researchers have studied the addition of enzyme preparations to reduce viscosity from potato mashes such as pectinase, cellulase and hemicellulase (Srichuwong et al. 2009; Srichuwong et al. 2012), and xylanase (Zhang et al. 2010), in order to disrupt the cell-wall matrix. Also, VHG technology can show incomplete fermentation, since the yeast cells are exposed to several stresses (high concentration of dissolved solids which increases external osmotic pressure, high ethanol concentration can be toxic to the cells) (Pradeep & Reddy 2010; Reddy & Reddy 2006).

Fresh sweet potato contains high water content. The drying process of this material is an aspect to be studied to optimize its transport, storing and processing. The use of flour of sweet potato would allow working with higher sugar concentration during the fermentation than fresh sweet potato without the addition of water. In this case, it should be assessed the energy saving of manipulating lesser amount of material, the handling of high viscous material, the extra cost of drying and the effect of drying on the performance of the process (conversion of starch to fermentable sugars) (Moorthy 2002).

The conventional process for bioethanol production from starch-based materials includes the conversion of starch into fermentable sugars which generally takes place in two enzymatic steps: liquefaction using thermal-stable alpha-amylase and saccharification by addition of amyloglucosidase (AMG). Most studies of starch hydrolysis use enzymes, temperature conditions and reaction times which have been done for grains, such corn. The starch of sweet potatoes is considered more complex than cereal starches, making it more challenging to hydrolyze into fermentable sugars. Besides, the digestibility of starch by enzymes varies among different cultivars (Duvernay et al. 2013; Moorthy 2002; Srichuwong et al. 2005). Yet there is still a need to establish a more defined biologically based approach to sweet potatoe starch conversion and evaluate the enzymes and processing conditions suitable for effective fermentable sugar production (Duvernay et al. 2013). The sweet potatoes used in this article has biomass yields of 10 t/ha (dry basis), higher value than cultivated varieties for human consumption which presented an average yield of up to 4.7 t/ha in Uruguay (http://www.mgap.gub.uy/portal/hgxpp001.aspx?7,5,659,O,S,0,MNU;E;27;8;MNU). No experimental information is available on the response of this variety of sweet potatoe to enzymatic saccharification and fermentation, including the use of high solid to liquid ratios.

The sweet potato used in this work (*Ipomoea batatas* K 9807.1) was identified as a sustainable crop for fuel bioethanol production based on both its favourable energy balance and the net GHG emission reduction, evaluated on a life cycle analysis conducted for local conditions in Uruguay (Carrasco-Letelier et al. 2013). It was developed as culture for bioenergy purposes on the basis of its high starch yields. This sweet potato variety had significantly reduced its attractiveness as a food crop when compared to other conventional food cultivars. The main aim was to study the two-step enzymatic hydrolysis of the sweet potato starch and the simultaneous saccharification and ethanol fermentation (SSF) of fresh and dried sweet potato (flour) by using mashes of different dry matter to water ratios. The drying effect on the integrity of starch and sugars, and their susceptibility to the hydrolysis after drying was also evaluated.

## Materials and methods

### Raw material, enzymes and microorganism

A sweet potato variety (*Ipomoea batatas* K 9807.1) was provided by INIA, Las Brujas, Canelones, Uruguay. To prepare a mash of fresh material, it was crushed into small pieces using a blender. The sweet potato flour was prepared by chipping the raw material and dried at 55°C until about 8% moisture content. Then it was milled to a mean particle size of 0.4 mm. Table 1 shows the sweet potato composition. The differences in the starch and free sugars content between the fresh sweet potato and the flour were due to the high variability in the composition of the original raw feedstock material. However, the total sugars expressed as glucose equivalents were similar for the two materials: 75.0% and 77.0% w/w of dry matter, for fresh sweet potato and flour respectively.

**Table 1:** **Sweet potato composition**

Sweet potato	Water content (%)	Free sugars (% w/w db)			Starch (% w/w db)	Total sugars in glucose equivalent (% w/w db)	Fiber (% w/w db)	Proteins (% w/w db)	Lipids (% w/w db)	Ash (% w/w db)
		Glucose	Fructose	Sucrose						
Fresh	73.1 ± 0.1	2.4 ± 2.1	2.6 ± 1.6	8.0 ± 0.4	55.5 ± 1.8	75.0 ± 6.1	1.0 ± 0.1	3.5 ± 0.8	0.4 ± 0.1	4.1 ± 0.3
Flour	7.7 ± 0.0	2.1 ± 0.2	1.6 ± 0.1	15.8 ± 0.6	51.1 ± 3.7	77.0 ± 5.0	3.0 ± 0.3	6.6 ± 1.5	1.8 ± 0.5	2.7 ± 0.1

db: dry base.

Total sugar in glucose equivalent was calculated as the sum: 1.11 × starch + glucose + fructose + 1.05 × sucrose.

The starch hydrolysis was performed using commercial enzymes: α-amylase (Liquozyme® SC, Novozymes) and amyloglucosidase (AMG) (Spirizyme® Fuel, Novozymes), a gift from Novozymes, Brazil. The activity of the enzymes was determined. One α-amylase unit (AAU) was defined as the amount of enzyme required to produce 0.1 g of reducing sugars expressed as glucose per minute. The α-amylase activity was 150 AAU/mL, using a solution of 1% of potato starch (SIGMA) in 1 M citrate buffer, gelatinized for 15 minutes at 90°C, pH 5.7-5.9, and 82°C-86°C. For the AMG, one AMG unit (AMGU) was defined as the amount of enzyme required to produce 0.05 g of reducing sugars expressed as glucose per minute. The AMG activity was 1000 AMGU/mL, using a solution of 2% of maltose (SIGMA) in 1 M citrate buffer, pH 4.0, and 60°C. The enzymatic activity was checked regularly.

Dry commercial baking yeast, *Saccharomyces cerevisiae* (Fleischmann) was used for the fermentation. The inoculum was prepared by adding 28 g of sweet potato (dry base) in a 500 mL Erlenmeyer flask containing 300 mL of distilled water. The medium was supplemented with salts: (NH_4_)_2_SO_4_ 0.24 g/L and MgSO_4_.7H_2_O 0.12 g/L. The pH was adjusted to 5.8, then 5.4 μL of α-amylase per gram of dry raw matter was added. It was maintained at 86°C during 90 min. The mash was cooled to 60°C, the pH adjusted to 4.0, then 5.4 μL of ΑΜG per gram of dry raw matter were added. It was kept at 60°C for 30 min. The pH was adjusted to 4.5, pasteurized at 100°C for 30 min, and inoculated with 5 g dry baking yeast. The culture was incubated in an orbital shaker at 30°C and 150 rpm for 12 h.

### Drying assays

Fresh sweet potato roots were cleaned and crushed into small pieces using a blender. One kg of the mash was dried in a tunnel dryer at operating conditions: 55°C or 95°C (± 3°C) and 0.5 m/s air velocity. The dried material was milled using a laboratory disk mill DLFU (Bühler). Starch, free sugars (glucose, fructose and sucrose) and moisture content were determined before and after drying. From 3 to 6 replications of each assay were performed. Hydrolysis assays were performed using the flour obtained under the optimized experimental conditions found for fresh sweet potato and flour. 300 mL of sweet potato mash with a dry matter to water ratio (w/v) of 1:5 was prepared in a 500 mL-Erlenmeyer flask, and then gelatinized at 90°C. The pH was adjusted to 5.8, then 5.4 μL of α-amylase per gram of dry raw matter was added to the mash. It was maintained at 86°C for 90 min under agitation. The mash was cooled to 60°C and the pH adjusted to 4.0. Then, 5.4 μL of AMG per gram of dry raw matter was added. The mash was maintained at 60°C for 30 min under agitation. At least three replications of each assay were performed.

### Gelatinization

The gelatinization assays were performed for a dry matter to water ratio (w/v) of 1:5 at 90°C, 100°C and 121°C using both fresh sweet potato and flour. 300 mL of sweet potato mash was prepared in a 500 mL-Erlenmeyer flask. The pH was adjusted to 5.8 and the mash was kept for 15 min at the temperature studied. At least 2 replications of each assay were performed.

### Liquefaction

The hydrolysis assays were performed for a dry matter to water ratio (w/v) of 1:5 using both fresh sweet potato and flour. Assays were performed with and without previous gelatinization. 300 mL of sweet potato mash was prepared in a 500 mL-Erlenmeyer flask. The pH was adjusted to 5.8. The reaction started by adding 5.4 μL of α-amylase per gram of dry raw matter. The mash was maintained at 86°C under agitation. The reaction was stopped with 40% trichloroacetic acid or 0.06 N NaOH and immersion in an ice batch at different times. From 2 to 10 replications of each assay were performed.

### Saccharification

After the starch liquefaction step, the sweet potato mash was cooled to 60°C and the pH adjusted to 4.0. Then, 5.4 μL of AMG per gram of dry raw matter was added. The mash was maintained at 60°C under agitation. The reaction was stopped with 0.06 N NaOH at different times. From 2 to 10 replications of each assay were performed.

### Simultaneous saccharification and fermentation (SSF)

SSF were performed using both fresh sweet potato and flour for different dry matter to water ratios (w/v). The ratios studied were 1:2.2 (corresponding to the fresh sweet potato without addition of water), 1:5 and 1:8 for fresh sweet potato, and 1:2, 1:3, 1:5 and 1:8 for sweet potato flour. The assays for ratios of 1:5 and 1:8 were performed using 500 mL-Erlenmeyer flasks containing 300 mL of sweet potato mash. Due to the high viscosity of the material, for ratios of 1:2, 1:2.2 and 1:3, the assays were conducted in 250 mL-Erlenmeyer flasks containing 150 mL sweet potato mash. In this case, the whole content of the flasks was used for the analyses.

The sweet potato mash was prepared by adding the right amount of water to the material (crushed fresh sweet potato or flour) in order to prepare a given dry matter to water ratio. The pH was adjusted to 5.8, then 5.4 μL of α-amylase per gram of dry raw matter was added to the mash. The mash was kept at 86°C for 90 min under agitation. It was cooled to 30°C, the pH adjusted to 4.5 and pasteurized at 100°C for 30 min. It was inoculated with *Saccharomyces cerevisiae* to an initial cell concentration of 1×10^8^ cell/mL and 5.4 μL of AMG per gram of dry raw matter was added. It was incubated in an orbital shaker at 100 rpm and 30°C. At least 2 replications of each assay were performed.

### Analytical methods

Sugars (sucrose, glucose, fructose and maltose), ethanol and glycerol concentrations were determined using a HPLC (Shimadzu, Kyoto, Japan) equipped with a Shodex SUGAR KS-801 column, or a Phenomenex Rezex RPM-Monosaccharide column, and a refractive index detector (RID-10A). The total sugar content in mashes was expressed in glucose equivalents (glucose + fructose + 1.05 × sucrose + 1.05 × maltose). The total sugar in raw matter was expressed in glucose equivalent as the sum: 1.11 × starch + glucose + fructose + 1.05 × sucrose.

The reducing sugar content was determined using the DNS technique using glucose as standard (Miller 1959).

Starch content was enzymatically determined by NREL analytical procedure (Sluiter & Sluiter 2005), proteins by Kjeldahl, lipids by Soxhlet method, fiber and ashes by AOAC methods. The moisture content was determined by drying at 60°C. Cellular concentration was determined by counting in a Neubauer chamber. Methylene blue staining was used to discriminate live and dead cells.

The viscosity profile during gelatinization and liquefaction of sweet potato flour mashes was determined using a starch cell in a rheometer (Anton Paar Physica MCR 301).

### Statistical analyses

Analyses of variance (ANOVA) of the data were performed for starch hydrolysis percentage using KaleidaGraph™, Synergy Software. Differences between means were considered significant when *p* ≤ 0.05.

## Results and discussion

### Gelatinization

**Figure 1:**
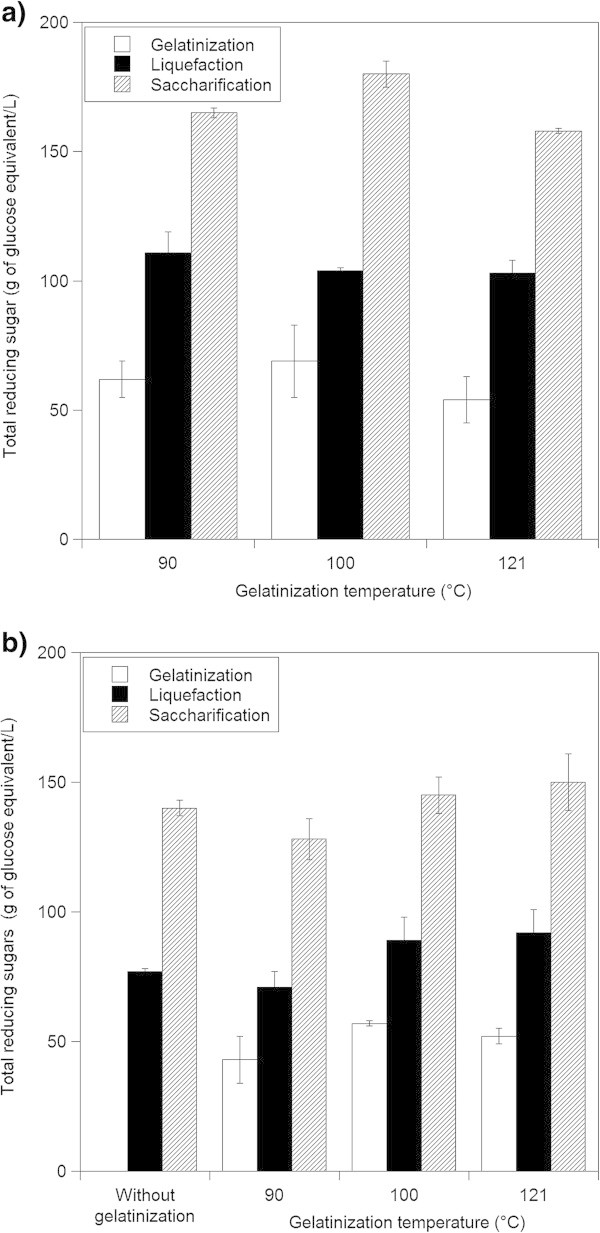
**Reducing sugar concentration (expressed as grams of glucose equivalent/L) after the gelatinization, liquefaction and saccharification processes. a)** Fresh sweet potato and **b)** flour. Dry matter to water ratio (w/v) of 1:5. Results are mean of 2 to 10 replications.

The heating step generated a highly viscous paste for the three temperatures studied (90°C, 100°C and 121°C). A better homogenization of the mash was observed with increasing temperature. Gelatinization would allow enzymes to penetrate easily into starch structures contributing to a more efficient reaction (Delgado et al. 2009; Hansen et al. 2008). After the gelatinization step, mean reducing sugar values of 62, 69 and 54 g glucose equivalent/L for fresh sweet potato and 43, 57 and 52 g glucose equivalent/L for flour were found for 90, 100 and 121°C respectively (Figure 1). The starch hydrolysis percentage was in the range of 47-61% and 34–45% for fresh material and flour respectively, without enzyme addition.

The reducing sugar concentration found after gelatinization without enzyme addition, was particularly high. According to the free soluble sugar content of the raw material, only 7 to 9 g of glucose equivalent/L should be found in the sweet potato mash. The high sugar content observed, suggests that the heat treatment produced a partial starch hydrolysis.

### Liquefaction

The liquefaction step involves the partial starch hydrolysis by the addition of the α-amylase at high temperature. High values of hydrolysis percentages were found after this process (in the range of 78% - 80% and 61% - 74% for fresh sweet potato and flour respectively). Figure 1 shows the sugar content after the liquefaction step.

The liquefaction was studied under the following conditions: (a) a gelatinization step was performed before the addition of the α-amylase at 90°C, and (b) without the gelatinization as a separate step (the enzyme was added before heating the sample to the liquefaction temperature, 86°C). No significant difference (*p* ≤ 0.05) was found for the starch hydrolysis percentages and reducing sugar concentration values for the two assays performed. After 90 min, similar values of reducing sugar concentration were found: 71 and 77 g of glucose equivalent /L for the two assays respectively (Figure 1).

**Figure 2:**
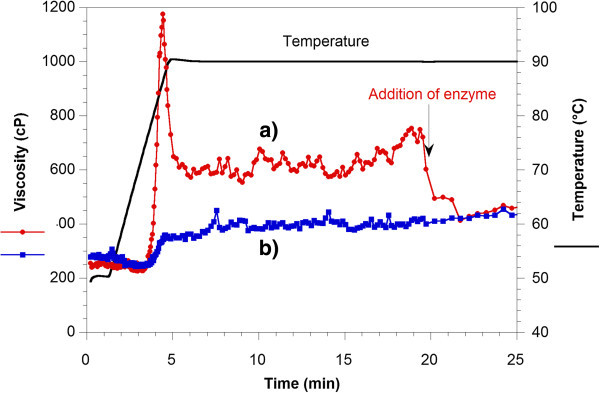
**Viscosity and temperature profiles for sweet potato flour mashes (dry matter to water ratio (w/v) 1:5) during the liquefaction process. (a)** The α-amylase was added to the sweet potato mash after gelatinization); **(b)** the α-amylase was added before heating. The α-amylase dose was 5.4 μL per gram of dry raw matter.

The preparation of starchy media for VHG fermentation produces mashes having very high viscosity, which are difficult to handle. The addition of α-amylase also reduces the starch-paste viscosity. Figure 2 shows the viscosity and temperature profiles during the gelatinization and liquefaction of the sweet potato flour mashes. These assays were performed for a dry matter to water ratio (w/v) of 1:5. The gelatinization temperature was 89°C, which corresponded to a viscosity peak of 1175 cP. Immediately after the addition of the α-amylase the viscosity decreased from 750 to 400 cP in few seconds. This fact demonstrated the high enzymatic activity of the α-amylase. For simultaneous gelatinization and liquefaction process, the viscosity profile did not present a peak as in the case of previous gelatinization before the addition of the enzyme. This would indicate that gelatinization was not observed, probably due to the rapid enzymatic action. The viscosity increased gradually reaching the value of 400 cP. Although the viscosity profiles for the two assays were very different, after the addition of the enzyme the viscosity values reached were very similar.

From the results found in this study, the gelatinization step before the addition of the amylase would not be necessary. It also allows working with sweet potato mashes with lower viscosity which improves the manipulation of the material especially for VHG conditions, in particular its homogeneity and transport, allowing the acquisition of more consistent results and reducing the energy consumption of the process.

### Saccharification

Saccharification of the sweet potato starch was assessed in assays where there was previous gelatinization to the liquefaction step and without the gelatinization step.

Figure 1 shows the final reducing sugar concentration found. For fresh sweet potato, a higher concentration was found at 100°C (165, 180 and 158 g/L were found for mashes gelatinized at 90°C, 100°C and 121°C respectively). For sweet potato flour mashes, final average reducing sugar concentrations of 128, 145 and 150 g/L were found for mashes gelatinized at 90°C, 100°C and 121°C respectively. These values corresponded to hydrolysis percentages close to 100% for both materials (fresh sweet potato and flour). Similar results were found for cassava (Shanavas et al. 2011). The total starch hydrolysis percentages reached was similar for all temperatures assayed. An ANOVA analysis (*p* ≤ 0.05) demonstrated that there was no significant difference for the temperatures studied.

Different times have been reported for the liquefaction and saccharification steps. Some researchers add AMG while the α-amylase is still acting (Mojović et al. 2006; Montesinos & Navarro 2000). This fact was based on the AMG activity, which can be inhibited by the presence of carbohydrates such as glucose. In this work, it was found that 90 min of the α-amylase action were sufficient to reach final starch hydrolysis percentages of 100% (after the addition of AMG).

**Figure 3:**
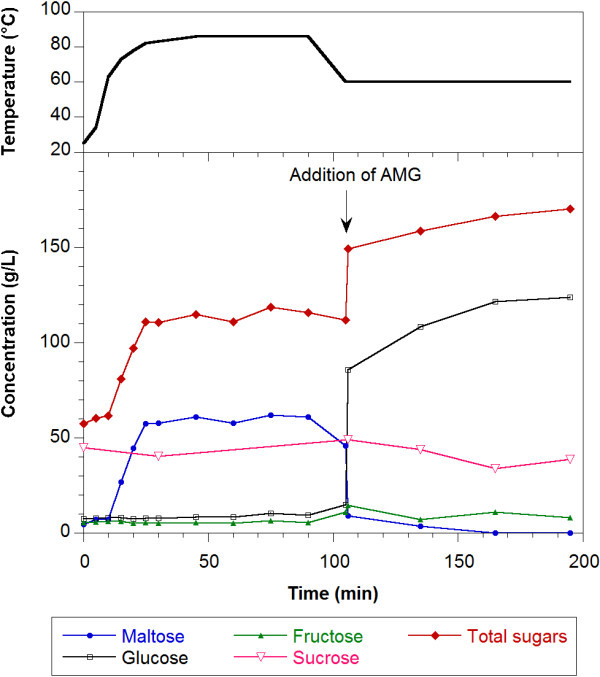
**Sugar and temperature profiles during the hydrolysis of sweet potato flour mashes without the gelatinization step, dry matter to water ratio (w/v) of 1:5.** The arrow indicates the addition of AMG. Total sugars are expressed as glucose equivalents.

Saccharification studies were also performed without the gelatinization step. Figure 3 shows the sugar profiles for the hydrolysis (liquefaction and saccharification steps). At 60 min of AMG action, a constant value of reducing sugar concentration was reached, which corresponded to the total starch hydrolysis. These facts permit to conclude that the gelatinization as a sole step before the addition of the enzymes was not needed to reach the complete hydrolysis.

### Effect of drying on sugar composition and hydrolysis

The effect of drying of sweet potato on the sugar composition and ethanol yield was studied. Table 2 shows the sugar composition before and after drying. No statistically significant loss of starch or free sugars was found after drying for the two temperatures studied. The weight loss during these assays agreed with the experimental water loss calculated from the moisture content data.

**Table 2:** **Sweet potato composition before and after drying**

Drying temperature (°C)	Drying time (h)	Free sugars (% w/w db)			Starch (% w/w db)	Total sugars in glucose equivalent (% w/w db)	Water content (%)
		Glucose	Fructose	Sucrose			
55	0	4.4 ± 0.1	2.9 ± 0.1	5.6 ± 0.1	60.4 ± 3.9	80.2 ± 4.6	68.4 ± 0.5
	28	3.6 ± 0.0	3.6 ± 0.0	4.6 ± 0.1	69.0 ± 1.4	88.6 ± 1.7	8.0 ± 1.1
95	0	3.9 ± 0.1	3.0 ± 0.1	5.0 ± 0.1	60.7 ± 2.1	79.5 ± 2.6	65.8 ± 1.5
	18	2.2 ± 0.0	3.3 ± 0.0	1.8 ± 0.1	59.7 ± 1.2	73.7 ± 1.4	7.8 ± 0.0

db: dry base.

Total sugar in glucose equivalent was calculated as the sum: 1.11 × starch + glucose + fructose + 1.05 × sucrose.

The enzymatic hydrolysis of the flour prepared at 55°C and 95°C were determined under the optimized conditions found for the fresh sweet potato and flour (discussed in gelatinization, liquefaction and saccharification sections). For both materials, 100% of total hydrolysis was reached.

### Simultaneous saccharification and fermentation (SSF)

SSF has been considered a good choice to reduce the osmotic pressure caused by high initial concentration of dissolved sugars in batch ethanol fermentation under VHG condition (Cao et al. 2011; Shen et al. 2011; Srichuwong et al. 2009; Zhang et al. 2011), and the feedback inhibition that the AMG could present by the presence of high concentrations of glucose (Cao et al. 2011; Mojović et al. 2006). In a SSF, the temperature and pH are more favorable for the yeast growth (~ 30°C) rather than for the AMG activity. Using this technology, the time and energy of the complete process can be reduced since the saccharification step separated from fermentation at temperatures above 50°C is eliminated (Zhang et al. 2010). Our results confirmed that the behavior of the SSF was better than SHF for fresh sweet potato using a dry matter to water ratio (w/v) of 1:5, in terms of sugars, ethanol and ethanol yield (data not shown).

The use of flour of sweet potato for ethanol production was assessed in order to ferment higher sugar concentration than fresh sweet potato without the addition of water (dry matter to water ratio (w/v) 1:2.2). Different dry matter to water ratios were studied using both fresh sweet potato and flour. Table 3 presents the results obtained.

**Table 3:** **Fermentation results for fresh sweet potato and flour at different dry matter to water ratios**

Sweet potato	Dry matter to water ratio (w/v)	Ethanol (g/L)	Glycerol (g/L)	Sugar conversion (%) (^*^)	Efficiency (%) (^¶^)	Productivity (g/Lh)	Industrial yield (L ethanol/t sweet potato dry base) (^†^)	Agroindustrial yield (L ethanol/ha) (^#,†^)
Fresh	1:2.2 (^‡^)	100 ± 11	9 ± 1	67 ± 7	92 ± 1	2.1 ± 0.3	320 ± 37	3170 ± 360
	1:5	63 ± 6	8 ± 1	88 ± 3	82 ± 4	2.6 ± 0.3	380 ± 34	3730 ± 330
	1:8	45 ± 5	8 ± 1	100 ± 0	84 ± 9	3.2 ± 0.4	490 ± 54	4790 ± 530
Flour	1:2	99 ± 1	12 ± 1	77 ± 2	79 ± 6	2.1 ± 0.1	305 ± 12	2990 ± 120
	1:3	97 ± 5	9 ± 1	100 ± 0	92 ± 5	2.7 ± 0.2	460 ± 22	4490 ± 220
	1:5	58 ± 1	7 ± 1	99 ± 2	90 ± 1	3.6 ± 0.2	425 ± 5	4170 ± 50
	1:8	38 ± 4	4 ± 1	99 ± 0	84 ± 8	2.5 ± 0.2	410 ± 41	4020 ± 400

(^*^) Sugar conversion based on the total sugar present in the raw material (fresh or flour).

(^¶^) Efficiency based on 0.511 g ethanol/g sugars as glucose.

(^†^) Calculated using the ethanol density at 20°C (0.7894 kg/L).

(^#^) Calculated based on an agriculture yield of 10 t/ha (dry matter) (Vilaró et al. 2009) and a distillation efficiency of 98%.

(^‡^) Fresh sweet potato without addition of water.

**Figure 4:**
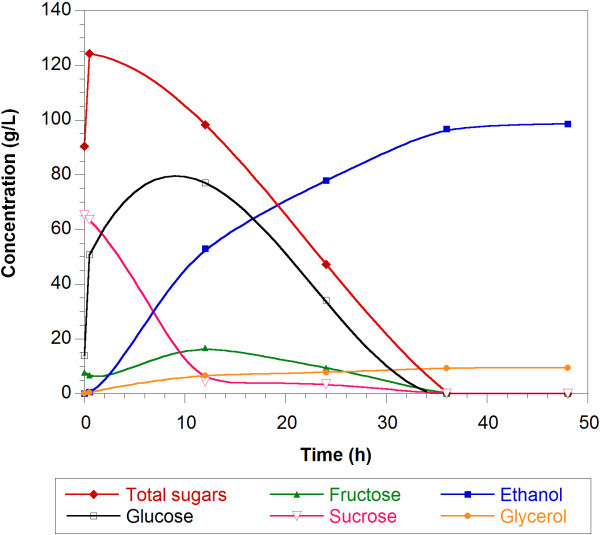
**SSF fermentation profiles using sweet potato flour, dry matter to water ratio (w/v) 1:3.** Total sugars are expressed as glucose equivalents.

**Figure 5:**
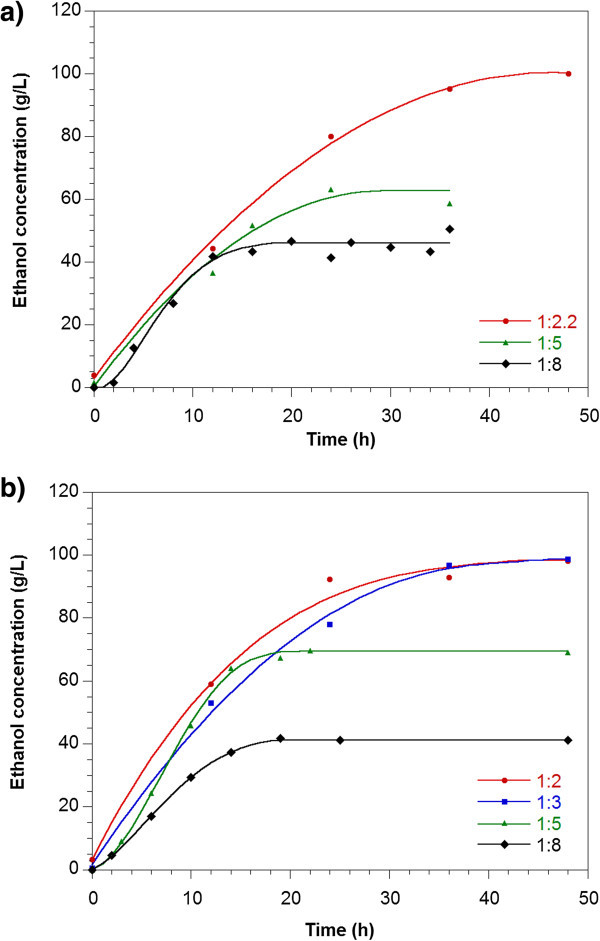
**Ethanol profiles for SSF for (a) fresh sweet potato and (b) sweet potato flour, for different dry matter to water ratios (w/v).** The ratio 1:2.2 corresponded to fresh sweet potato without addition of water.

Figure 4 shows typical fermentation profiles for sweet potato flour and a dry matter to water ratio (w/v) of 1:3. Although it was a SSF where the temperature was lower than the optimum for the AMG, the rate of hydrolysis was higher than the rate of glucose consumption by the yeast. At 36 h of fermentation, the sugar conversion was completed. Figure 5a and 5b show the ethanol profiles for fresh sweet potato and flour respectively.

The ethanol concentration and the fermentation time were greater for high dry matter content (Table 3). For fresh sweet potato, the sugar consumption was completed only for the dry matter to water ratio (w/v) of 1:8 (Table 3). For ratios 1:5 and 1:2.2, the sugar concentration was constant at 34 g/L and 107 g/L after 24 h and 48 h respectively. The maximum ethanol concentration reached was similar to that found for flour (close to 100 g/L).

For sweet potato flour, the ratios 1:8, 1:5 and 1:3 showed total sugar conversion; however for the ratio 1:2, the fermentation was not completed since after 48 h the total residual sugars remained constant at 72 g/L (stuck fermentation). The maximum ethanol concentration found using the baker yeast *Saccharomyces cerevisiae* was close to 100 g/L (98 and 97 g/L for dry matter to water ratio (w/v) of 1:2 and 1:3 respectively). It seems that higher ethanol concentrations than 100 g/L were toxic for this microorganism. It has been reported that the exposure to toxic levels of ethanol is the severest of the various stresses that the yeast cells experience during fermentation, particularly under VHG conditions (Zhao & Bai 2009). Since the ethanol tolerance was reported to be improved by proper supplementation of nutrients such as different sources of nitrogen, vitamins and metal ions to the media (Breisha 2010; Shen et al. 2012; Zhao & Bai 2009), the addition of nutrients may contribute to the increase of the final ethanol concentration under VHG conditions (high dry matter to water ratios). Breisha (2010) found complete consumption of 35% sucrose and 16% ethanol produced for a ratio of added nitrogen to sucrose of 5 mg/g of sucrose (the nitrogen as ammonium sulphate), addition of yeast extract, thiamine and air during the first hours of fermentation. However, many of the medium supplements used in laboratory research, such as amino acids, vitamins, sterols and unsaturated fatty acids, are too expensive to be used in the industry. Thus, ethanol-tolerant yeast would be needed for efficient fermentation (Pereira et al. 2011; Watanabe et al. 2010).

The final ethanol concentration was similar for fresh sweet potato and flour at the same dry matter to water ratio. For both materials used, the concentration of glycerol was in the range 4 to 9 g/L at the end of the fermentations. The glycerol concentration was higher for the higher dry matter to water ratios (higher solid concentration) as expected since the glycerol is produced by the cells as response to an osmotic stress, due to high sugar concentration (Pereira et al. 2011). The total cell concentrations were almost constant during all fermentations.

Agroindustrial yields up to 4800 L/ha (calculated based on ethanol produced, the real amount of sweet potato used in the experiment, crop yield (Vilaró et al. 2009) and a distillation and dehydration efficiency of 0.98) were observed. Such yields are very promising, since agroindustrial crops used for ethanol production in Uruguay, mainly sugar cane and grain sorghum, have yields of 3600 and 1800 L/ha, respectively (Carrasco-Letelier et al. 2013). Similar results were found by Jin et al. (2012).

## Conclusions

Drying of sweet potato neither affected the sugar content nor the starch enzymatic hydrolysis efficiency. The dry matter content of sweet potato mashes should be carefully selected to have high yields, high final ethanol concentrations and fast fermentations. Faster full sugar conversions were observed for high dry matter content of flour mashes. Higher dry matter content than that for fresh sweet potato, did not improve the final ethanol concentration. The availability of ethanol-tolerant yeasts might improve the performance. The sweet potato used is an attractive raw matter for fuel ethanol, since up to 4800 L ethanol per hectare can be obtained.
